# Fit-Tested N95 Masks Combined With Portable High-Efficiency Particulate Air Filtration Can Protect Against High Aerosolized Viral Loads Over Prolonged Periods at Close Range

**DOI:** 10.1093/infdis/jiac195

**Published:** 2022-05-10

**Authors:** Shane A Landry, Dinesh Subedi, Jeremy J Barr, Martin I MacDonald, Samantha Dix, Donna M Kutey, Darren Mansfield, Garun S Hamilton, Bradley A Edwards, Simon A Joosten

**Affiliations:** Department of Physiology, School of Biomedical Sciences and Biomedical Discovery Institute, Monash University, Melbourne, Victoria, Australia; School of Biological Sciences, Monash University, Clayton, Victoria, Australia; School of Biological Sciences, Monash University, Clayton, Victoria, Australia; Monash Lung, Sleep, Allergy and Immunology, Monash Health, Clayton, Victoria, Australia; Monash Nursing and Midwifery, Monash University, Clayton, Victoria, Australia; Monash Nursing and Midwifery, Monash University, Clayton, Victoria, Australia; Monash Lung, Sleep, Allergy and Immunology, Monash Health, Clayton, Victoria, Australia; School of Clinical Sciences, Monash University, Melbourne, Victoria, Australia; Monash Partners–Epworth, Victoria, Victoria, Australia; Monash Lung, Sleep, Allergy and Immunology, Monash Health, Clayton, Victoria, Australia; School of Clinical Sciences, Monash University, Melbourne, Victoria, Australia; Monash Partners–Epworth, Victoria, Victoria, Australia; Department of Physiology, School of Biomedical Sciences and Biomedical Discovery Institute, Monash University, Melbourne, Victoria, Australia; Turner Institute for Brain and Mental Health, Monash University, Melbourne, Victoria, Australia; Monash Lung, Sleep, Allergy and Immunology, Monash Health, Clayton, Victoria, Australia; School of Clinical Sciences, Monash University, Melbourne, Victoria, Australia; Monash Partners–Epworth, Victoria, Victoria, Australia

**Keywords:** aerosols, personal protective equipment, ventilation, air filtration

## Abstract

**Background:**

Healthcare workers (HCWs) are at risk from aerosol transmission of severe acute respiratory syndrome coronavirus 2. The aims of this study were to (1) quantify the protection provided by masks (surgical, fit-test_FAILED_ N95, fit-test_PASSED_ N95) and personal protective equipment (PPE), and (2) determine if a portable high-efficiency particulate air (HEPA) filter can enhance the benefit of PPE.

**Methods:**

Virus aerosol exposure experiments using bacteriophage PhiX174 were performed. An HCW wearing PPE (mask, gloves, gown, face shield) was exposed to nebulized viruses (10^8^ copies/mL) for 40 minutes in a sealed clinical room. Virus exposure was quantified via skin swabs applied to the face, nostrils, forearms, neck, and forehead. Experiments were repeated with a HEPA filter (13.4 volume-filtrations/hour).

**Results:**

Significant virus counts were detected on the face while the participants were wearing either surgical or N95 masks. Only the fit-test_PASSED_ N95 resulted in lower virus counts compared to control (*P* = .007). Nasal swabs demonstrated high virus exposure, which was not mitigated by the surgical/fit-test_FAILED_ N95 masks, although there was a trend for the fit-test_PASSED_ N95 mask to reduce virus counts (*P* = .058). HEPA filtration reduced virus to near-zero levels when combined with fit-test_PASSED_ N95 mask, gloves, gown, and face shield.

**Conclusions:**

N95 masks that have passed a quantitative fit-test combined with HEPA filtration protects against high virus aerosol loads at close range and for prolonged periods of time.

## Teaser

Only a fit-tested N95 mask provided protection against virus aerosol; surgical masks and non-fit-tested N95 masks were not superior to no-mask control. The combination of air purification and fitted N95 mask provided protection against very high virus aerosol load.

**(See the Editorial Commentary by Klompas and Rhee on pages 191–4.)**

Virus-laden aerosols can remain suspended in the air for prolonged periods of time and travel large distances while remaining infectious [1, 2]. Epidemiological evidence from previous severe acute respiratory syndrome outbreaks highlight the importance of aerosol transmission [3, 4]. Nosocomial infection risk was demonstrated to be greatest in the setting of “aerosol-generating procedures” [5], which informed enhanced respiratory protection recommendations for healthcare workers (HCWs) caring for patients with coronavirus disease 2019 (COVID-19) undergoing such procedures. However, recent work has shown that HCWs caring for patients not receiving aerosol-generating procedures contracted COVID-19 despite the use of surgical masks and personal protective equipment (PPE) [6–8]. Subsequent studies demonstrated that aerosols laden with infectious severe acute respiratory syndrome coronavirus 2 (SARS-CoV-2) are present in the rooms of COVID-19 patients in the absence of aerosol-generating procedures [9], likely because aerosols are self-generated by COVID-19 patients when they cough, talk, and breathe.

These findings led the Centers for Disease Control and Prevention (CDC) to recommend that HCWs wear an N95 (or higher-level respirator) and to optimize indoor air quality, including the deployment of portable high-efficiency particulate air (HEPA) filters when permanent air-handling systems cannot be feasibly improved [10]. Importantly, there is evidence that each measure in isolation (enhanced personal protection and enhanced environmental protection) may be inadequate. For example, pre–COVID-19 studies demonstrated that respiratory infections still occur in the context of N95 [11] and fit-tested N95 respirators [12]. Furthermore, a Cochrane review showed uncertainty for any benefit of N95 masks over surgical masks in protecting against respiratory illness [13]. Regarding environmental protection, COVID-19 can still be transmitted in outdoor (ie, optimally ventilated) settings [14] and airborne SARS-CoV-2 can still be detected in negative-pressure isolation rooms (≥12 air exchanges/hour) [15].

We aimed to examine 2 mechanistic questions regarding the effectiveness of personal PPE and air filtration to protect HCWs against virus aerosol: first, to quantify the degree of personal contamination with virus aerosol when wearing different types of masks (surgical, fit-test_FAILED_ N95), and a fit-test_PASSED_ N95 mask) in combination with face shield, gown, and disposable gloves; and second, to determine if the use of a portable HEPA filter enhances the benefit of PPE to protect the wearer against virus aerosol contamination.

## METHODS

### Bacteriophage PhiX174 Propagation and Titration

Bacteriophage PhiX174 was used as a nonhazardous model virus in all experiments. PhiX174 was propagated using bacterial host *Escherichia coli* C (ATCC 13706) in lysogeny broth. The bacteriophage was purified according to the Phage-on-Tap protocol [16]. A titer of 1–5 × 10^9^ plaque-forming units (PFU)/mL was obtained and diluted in 1× phosphate-buffered saline (PBS; Omnipur, Gibbstown, New Jersey). The bacteriophage titer was determined using the standard soft agar overlay method. A 10-mL bacteriophage lysate (10^8^ PFU/mL, a total of 10^9^ PFU) was aerosolized (via nebulizer) in all experiments. The choice of titer was determined by pilot sensitivity experiments [17, 18] with the aim of detecting a strong positive in our control condition (no PPE, no ventilation/filtration) from which relative reductions can be assessed.

### Simulated Virus Aerosol Exposure

**Figure 1.**
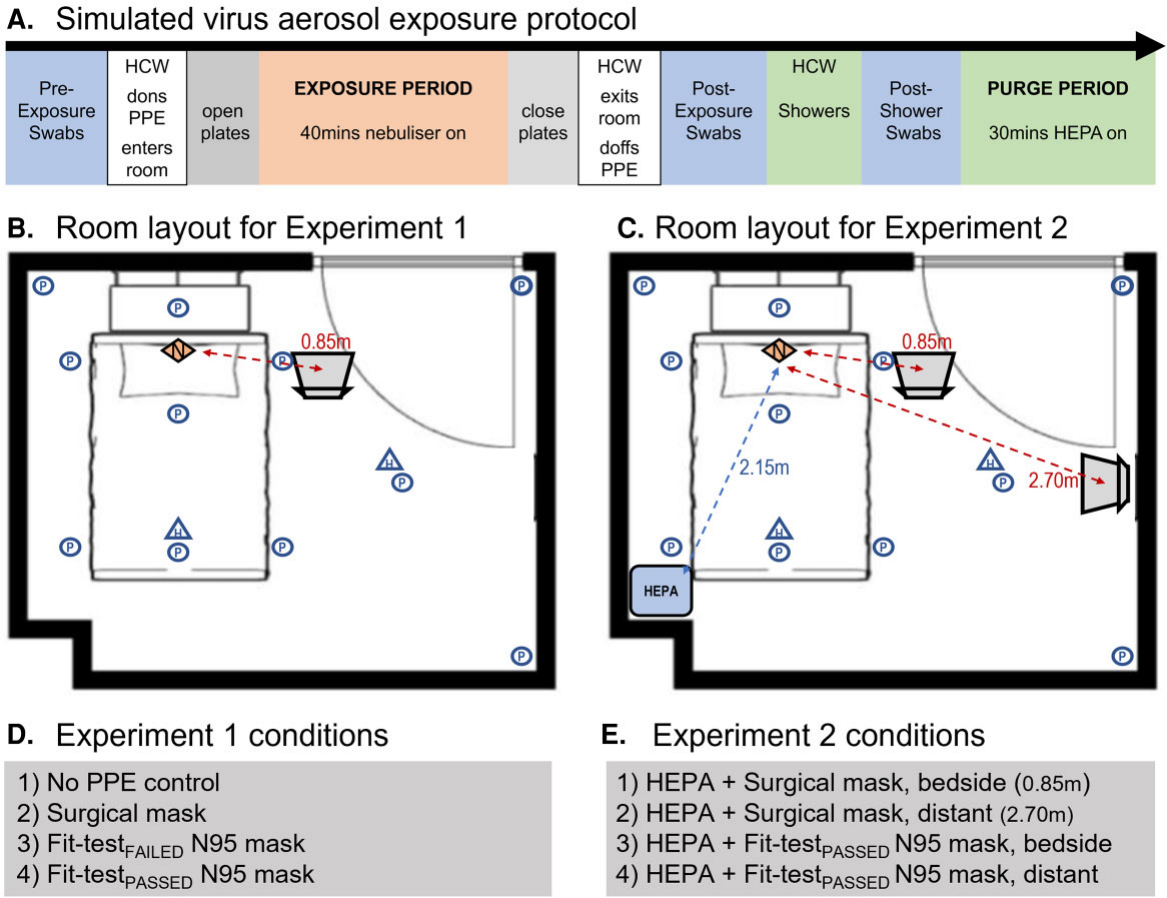
Experimental protocols. *A*, Experimental virus exposure experiments were performed with the same timed protocol. Preexposure skin and nasal swabs were collected prior to the HCW donning personal protective equipment (PPE) and entering the clinical room. Settle plates were opened and the HCW sat in the nominated position. The nebulizer containing the bacteriophage lysate was turned on remotely for 45 minutes (exposure period) after which settle plates were closed and the HCW exited the room and doffed PPE. Postexposure swabs were collected. The HCW then showered, and further postshower swabs were collected. A HEPA filter was run for 30 minutes following the exposure period to purge the room of virus aerosol. Control plates were deployed for 10 minutes to ensure no further virus settling. This protocol was then repeated with a different experimental condition. *B*, All experiments were performed in a clinical room with dimensions 4.0 × 3.25 × 2.7 m (volume = 35.1 m^3^) containing a bed and 1 chair. Eleven settle plates (circles) were positioned identically between both experiments. Two hanging plates (triangles) were hung at head height perpendicular to the floor. The nebulizer (diamond) was positioned at the head of the bed, with the exit point facing vertically. In experiment 1, the healthcare worker (HCW) was seated at the bedside, 0.85 m from the aerosol source for all conditions. *C*, For experiment 2, the high-efficiency particulate air (HEPA) filter was positioned at the foot and opposite side of the bed to the HCW, 2.15 m from the nebulizer. The HCW was positioned either at the same beside position as experiment 1 (0.85 m) or at a distanced location (2.70 m) from the nebulizer. *D*, Experiment 1 tested 4 mask conditions: no-PPE control, surgical mask, fit-test_FAILED_ N95 mask, and a fit-test_PASSED_ N95 mask. In each mask condition, participants also wore gloves, gown, and face shield. All 4 experimental conditions were completed in a single day in a randomized order. Replicate experiments/conditions were completed on subsequent days. *E*, Experiment 2 tested 4 mask/distance conditions: surgical mask at bedside, surgical mask at distant position, fit-test_PASSED_ N95 at bedside, and fit-test_PASSED_ N95 at distanced position. For this experiment the HEPA filter was run at the highest setting (470 m^3^/hour) for the entire exposure period.

A nebulizer (PARI Respiratory Equipment), positioned at the head of the bed, aerosolized the bacteriophage lysate within a simulated clinical room (dimensions: 4.0 × 3.25 × 2.7 m, volume = 35.1 m^3^; Figure 1). The Pari-PEP nebulizer produces a distribution of aerosol with a narrow particle size (3.42 ± 0.15 µm) [19]. However, to confirm the particle size generated by the nebulizer, we recorded particle mass concentration with a PurpleAir PA-II-SD (PurpleAir Inc) sensor for reference (Supplementary Figure 1)

A HCW wearing PPE remained seated in the room during nebulization (approximately 40 minutes), in 1 of 2 locations (Figure 1). The “bedside” location was positioned 0.85 m from the nebulizer, whereas the “distanced” location was at 2.70 m.

After exiting the room, the HCW doffed PPE according to a standardized protocol (see Supplementary Methods). Skin/nasal swabs quantified HCW contamination from viruses infiltrating PPE during the exposure period. Swab samples were collected by a single experimenter (S. A. L.). Swabs were immersed in 3 mL of 1× PBS in a test tube, and applied individually to 5 areas: forearms, neck, forehead, mouth/nose under the mask, and nostrils (rotated 360° 1–2 cm within the nasal vestibule) (Supplementary Figure 2). Swabs were then reimmersed in PBS and sealed within the test tube. Next, 1 mL of PBS (with swab immersed) was collected and quantified using standard soft agar overlay. The HCW then showered and performed a saline nasal rinse (Supplementary Table 1). Skin swabs were then repeated postshower.

Settle plates were used to quantify environmental contamination from virus aerosol [20, 21] Thirteen settle plates were positioned uncovered during each nebulization (Figure 1). Afterward, plates were sealed and incubated overnight at 37°C, and viral plaques were enumerated the following day. Quantification of PFU was performed by a single researcher blinded to experimental conditions (D. S.).

Given that this research involved no human or animal participants (HCWs were researchers), the Monash University Human Research Ethics committee exempted this protocol from ethics review.

### Experimental Protocols

*Experiment 1.* To assess the efficacy of PPE to protect against virus aerosol exposure, the HCW was seated at the bedside during nebulization. Virus counts from face/nasal swabs were used to assess the efficacy of the (1) surgical mask, (2) fit-test_FAILED_ N95 mask, and (3) fit-test_PASSED_ N95 mask. A gown, gloves, and face shield were worn in each condition. A no-PPE condition served as a control. All 4 mask conditions were tested on the same day in a randomized order using a computer-generated sequence. Each condition was replicated 5 times over 5 separate days.

*Experiment 2.* To assess the efficacy of combining multiple control measures, the same experimental paradigm was used with constant HEPA filtration. The HCW was seated either bedside (0.85 m from aerosol source) or at a distanced location (2.70 m from source), with the HEPA filter placed at the foot of the bed (Figure 1). The HCW wore either a surgical mask or a fit-test_PASSED_ N95 mask. All 4 conditions were tested on the same day in a randomized order and replicated 3 times over 3 separate days.

### Personal Protective Equipment

Experiments were performed with a single HCW (S. A. J.) wearing a gown (Virafree Isolation Gown, Jiangxi Fashionwind Apparel), gloves (Nisense nitrile gloves, Mediflex Industries), and face shield (PET Face Shield, Xamen Sanmiss Bags Co).

Three mask variants were tested:

- 3-ply surgical mask (OBE Premium face mask)
- Fit-test_FAILED_ N95 respirator (defined by failed quantitative fit test, fit factor < 100)

(BYD N95 Healthcare Particulate Respirator, National Institute for Occupational Safety and Health [NIOSH] approval number 84A-9279)

- Fit-test_PASSED_ N95 respirator (defined by passed quantitative fit test, fit factor = 194)

(3M Aura 1870A, NIOSH approval number 84A-5726)

Quantitative fit testing was performed via TSI PortaCount Fit Tester Model 8048, which measures the concentration of particles in the ambient air relative to within a respirator to calculate a fit factor.

Before experiments, masks were individually fit checked (qualitatively) and form-fitted by the wearer to optimize fit. Breathing was at rest and predominantly nasal with periods of oral breathing to check/confirm mask fit. The HCW was clean shaven prior to each experiment to reduce mask/beard interactions.

After aerosol exposure, PPE was doffed in a room separated from the clinical room by a corridor and 4 sealed doors. The doffing room had continuous HEPA filtration (5 air exchanges/hour). Doffing was videoed and examined independently by 2 expert nurses (S. D., D. M. K.) to ensure doffing procedure compliance (Supplementary Methods).

### Clinical Room

The clinical room had a double-sealed soundproofed/insulated door. To control airflow patterns, ceiling vents were taped shut and heating/cooling appliances were switched off. The room temperature (median, 23.3°C [min–max, 20.8°C–26.6°C]), humidity (median 42% [min–max, 32%–54%]), and barometric pressure (median, 993.8 mm Hg [min–max, 999.3–1018 mm Hg]) were well controlled during experimental procedures. Individual measurements for each experiment are available via the Supplementary Results.

### HEPA Filtration

The IQAir HealthPro250 was used at its highest clean air delivery rate 470 m^3^/hour, which based on the room volume, achieved 13.4 volume-filtrations/hour. This device was run for 30 minutes (∼6.7 filtration exchanges) after nebulization to remove bacteriophages before repeating experiments. This was then confirmed by deployment of control plates.

### Data Analysis

Viable viruses were quantified by counting the number of viruses from swabs and settling plates. Virus counts >200 were considered too many to count (TMTC) and rated using an ordinal visual rating scale (+, ++, +++, ++++), with TMTC++++ indicating complete lysis of the bacterial host. For graphing/analysis, TMTC ratings were given values of 200–230. Wilcoxon, Mann-Whitney *U*, or Friedman tests (χ^2^_Friedman_) with post hoc comparisons (Dunn test) were used to compare virus counts between conditions.

## RESULTS

### Experiment 1: Efficacy of PPE to Mitigate Healthcare Worker Exposure From Virus Aerosol

Settle plates confirmed substantial virus contamination of surfaces in the clinical room across all experimental conditions (Supplementary Figure 3).

**Figure 2.**
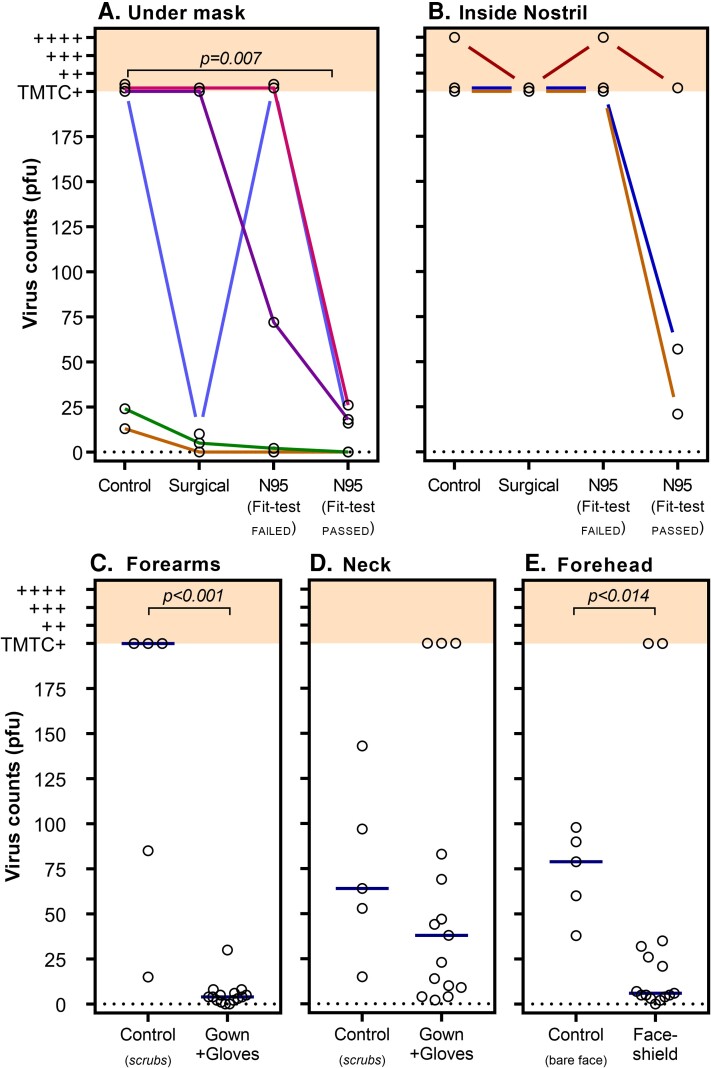
The effect of personal protective equipment (PPE) on virus plaque counts. Virus counts recovered from skin swabs (open circles, y-axis) and the mitigating effect of differing types of PPE (conditions described on x-axes) are shown. Virus counts were quantified as plaque-forming units (PFU) as previously described [16]. Virus counts >200 were considered too many to count (TMTC) and were rated using an ordinal (+, ++, +++, ++++, shown in shading) visual rating scale. *A*, Virus counts measured around mouth/nose underneath mask. Compared to the non-masked control condition, virus counts were found to be significantly lower when a fit-test_PASSED_ N95 mask was worn (χ^2^_Friedman_ = 9.075, *P* = .017). Colored lines represent data collected on same day (in randomized order). While there is distinct variability in virus counts within conditions, data collected on the same day (with same bacteriophage titer) show consistent trends of reduced virus counts for the fit-test_PASSED_ N95 mask. *B*, Virus counts were the highest when measured from inside the nostril. There was a trend (*P* = .058) for a fit-test_PASSED_ N95 mask to reduce virus counts. However, a surgical mask and fit-test_FAILED_ N95 did not appear to mitigate virus exposure. *C*, Virus counts were substantially lower on forearms/back of the hands when a gown and gloves were worn compared to a control condition in which no PPE (only scrubs) was worn (*P* < .001). *D*, Virus counts on the neck were not significantly reduced by a gown with an exposed neck, compared to no-PPE control condition (*P* = .297). *E*, Virus counts recovered from forehead swabs were significantly lower when wearing a face shield compared to the no-PPE control condition (*P* = .014). Bars (shown in panels *C–E*) represent median values.

Virus counts recovered from skin under the mask significantly differed by mask type (χ^2^_Friedman_ = 9.08, *P* = .017; Figure 2*A*); however, only the fit-test_PASSED_ N95 mask resulted in significantly lower virus counts compared to the no-mask control (*P* = .007, fit-test_PASSED_ N95 mask vs control). Given the very high and variable virus counts recovered from the skin under the mask, skin swabs taken from within the nostril were introduced on the third experiment day (ie, 3 repetitions available). Virus counts from inside the nostril (Figure 2*B*) were consistently high for control, surgical, and fit-test_FAILED_ N95 mask conditions. There was a trend for the fit-test_PASSED_ N95 mask to reduce virus counts on nasal swab (*P* = .058, fit-test_PASSED_ N95 vs control); however, positive virus counts were still recovered on all tests.

There were highly variable virus counts within conditions, particularly under the mask (Figure 2). This variability was most notable between testing days, likely driven by small day-to-day differences in the bacteriophage titer, whereas within-day (in which all mask types were compared in a randomized order, Figure 2*A* and 2*B*), there were consistent trends suggesting the fit-test_PASSED_ N95 mask always performed superior to the control condition. Similarly, surgical and fit-test_FAILED_ N95 masks were largely superior (excepting 1 case) compared to control.

To assess the efficacy of gloves, gown, and face shields to reduce virus counts recovered from the body, data were combined across mask conditions and compared to the no-PPE control (Figure 2*C*–*E*). A gown and gloves substantially reduced virus counts on the forearms/hands compared to the no-PPE control (U = 1, *P* < .001). Viruses were detectable on all neck samples and there was no significant difference between the no-PPE control (note the gown used does not cover the neck; U = 25, *P* = .297). Virus counts measured from forehead swabs were significantly reduced with the face shield compared to the no-PPE control (U = 10, *P* = .014).

### Experiment 2: Efficacy of Combining PPE, HEPA Filtration, and Distancing

Settle plates demonstrated substantial virus counts during nebulization despite the presence of the HEPA filter. Plates closest to the aerosol source (Figure 1, plates 4 and 5) demonstrated the highest virus counts. Overall, virus counts from plates were significantly lower with HEPA filtration compared to virus counts on plates from experiment 1 (Supplementary Figure 5).

**Figure 3.**
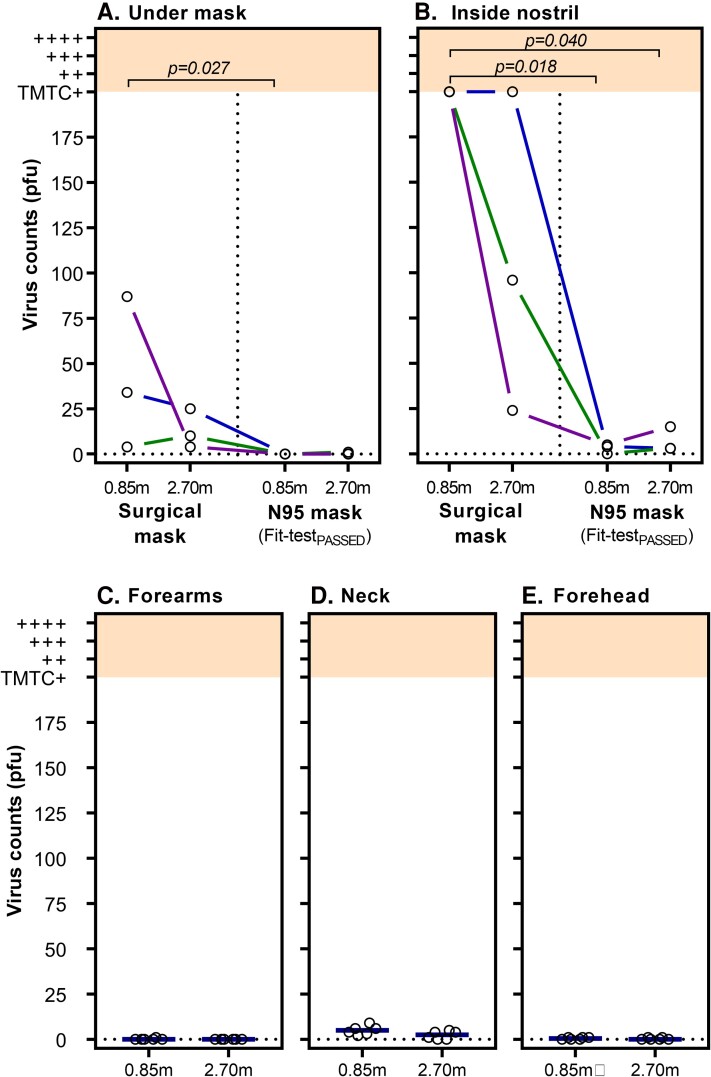
High-efficiency particulate air (HEPA) filtration combined with personal protective equipment (PPE) and distance on virus plaque counts. Virus counts from skin swabs (open circles, y-axis) are shown at 0.85 m (ie, bedside) and 2.5 m (distanced) locations (x-axis). Virus counts were quantified as plaque-forming units (PFU). Colored lines connect data points collected on the same day (same exact bacteriophage titer). Virus counts >200 were considered too many to count (TMTC) and were rated using an ordinal (+, ++, +++, ++++, shown in shading) visual rating scale. A HEPA filter set to a clean air filtration rate of 470 m^3^/hour (equivalent to 13 exchanges/hour) is present in all conditions. *A*, Virus counts recovered from under the mask were significantly lower with a fit-test_PASSED_ N95 mask. *B*, Virus counts from inside nostril were substantially higher for the surgical mask compared to the fit-test_PASSED_ N95 mask. Combining HEPA filtration and PPE (gown, gloves, and face shield) resulted in very low virus counts on forearms (*C*), neck (*D*), and forehead (*E*); however, the neck was the least protected body site, likely due to no coverage provided by the gown. Bars (shown in panels *C–E*) represent median values.

Virus counts were on average lower across swab locations and conditions compared to experiment 1 (Figure 3). Virus counts from under the mask significantly differed between conditions (χ^2^_Friedman_ = 7.93, *P* = .028; Figure 3*A*) and were higher at bedside with a surgical mask compared to the fit-test_PASSED_ N95 mask at bedside (*P* = .027). There was trend suggesting the fit-test_PASSED_ N95 mask at bedside outperformed the surgical mask at distance (*P* = .058). Notably, virus counts were near zero for the fit-test_PASSED_ N95 mask at both distances.

Consistent with experiment 1, virus counts from nasal swabs were higher than all other swab sites and varied substantially via mask type and distance (χ^2^_Friedman_ = 7.97, *P* = .017; Figure 3*B*). Nasal virus counts under a surgical mask were consistently TMTC and significantly higher than the fit-test_PASSED_ N95 mask at both bedside (*P* = .017) and distanced (*P* = .040) positions. While nasal swab virus counts were lower with the fit-test_PASSED_ N95 at bedside compared to the surgical mask at distance (*P* = .082), this was not statistically significant, similar to the results for swabs under the mask. Virus counts did not significantly differ based on distance for surgical masks (*P* = .527, surgical mask bedside vs distance), or for the fit-test_PASSED_ N95 mask (*P* = .752, fit-test_PASSED_ N95 bedside vs distance) likely to due to floor effect.

The combination of PPE (gown, gloves, face shield) and HEPA filtration resulted in very low virus counts on the forearms and forehead, regardless of distance from aerosol source (highest virus count recovered from swabs in all experiments was 1 PFU). The neck site had low virus counts (median [interquartile range], 5 [2.75–7.75] for bedside; 2.5 [0–4.25] for distanced), but were consistently higher compared to forehead swabs. The difference between bedside and distanced was not significant (*P* > .99, neck site, bedside vs distanced).

### Effect of Showering on Virus Counts

Showering between experimental conditions reduced virus counts to near zero on postshower skin and nostril swabs. The reduction in virus counts achieved with showering was significant for each swab site (Supplementary Figures 6 and 7).

## DISCUSSION

Our study is the first to conduct live virus aerosol experiments to systematically examine HCW virus contamination and the interaction between virus aerosol, PPE, and air filtration using a portable HEPA filter. We found that the combination of a N95 mask that passed a quantitative fit-test and portable HEPA filter provided near complete protection against high viral aerosol loads at close range for prolonged periods of time. Critically, surgical masks provided inadequate protection against skin and upper airway contamination, even when combined with HEPA filtration and at distances of 2.70 m. In light of aerosol transmission of SARS-CoV-2 and the emergence of more transmissible variants of concern, our findings have immediate and broad implications for the protection of HCWs.

Clinical evidence for the superiority of N95 respirators over other mask types in protecting against SARS-CoV-2 infection is mixed [22–24]. Our data identify 2 possible mechanistic reasons for mixed clinical signals: (1) mask fit and (2) fallibility at high viral load. Approved N95 respirators perform to a filtration standard that protects against particles to the nanometer range [25]. Importantly, previous reports of surgical and N95 mask penetration properties show that peripheral leak is more important than the filtering properties of the mask material [26]. Gaps between the face and mask provide low resistance points for airflow to circumvent the (higher resistance) mask filter. Poorly fitting masks allow significant airflow through these gaps into which virus-laden aerosol can infiltrate. Our study demonstrates that a N95 mask that passed a quantitative fit-test reduces skin and nasal virus aerosol contamination compared to fit-test_FAILED_ N95 and surgical masks. Importantly, both the fit-test_FAILED_ N95 and surgical masks were fit checked by the wearer at time of each application to ensure the best possible fit for that specific mask during each condition. A fit-test_PASSED_ N95 was the only condition that proved superior to control (no mask). It is also noteworthy that the fit-test_FAILED_ N95 was appropriately sized and had no external qualitative indicators of poor fit and was form-fitted on each application per protocol. Despite this, the fit-test_FAILED_ N95 performed with similar efficacy as surgical mask, again highlighting the critical importance of mask fit. These findings reinforce the necessity of quantitative fit testing of N95 respirators for all forward-facing HCWs, a process that is not universal practice and that relies on available mask supply [27]. However, in our study, even with the best-fitting N95 mask there was still virus aerosol contamination of the nose after 40 minutes’ exposure to high virus aerosol load at close range in the absence of HEPA filtration (Figure 2*B*).

Patients with airborne infectious diseases are typically cared for in negative-pressure rooms that are designed to facilitate 12 air exchanges/hour. However, such rooms are rapidly depleted/overwhelmed in an infectious respiratory disease pandemic. An alternative method is to filter the air within a patient room with portable HEPA filters. The CDC recommends that HEPA filters could be used in lieu of negative-pressure rooms while hospital facilities are being renovated/repaired [28]. This idea gained traction throughout the COVID-19 pandemic as airborne spread of SARS-CoV-2 became widely acknowledged. Although clinical trials are lacking, there are several experiments that demonstrate the efficacy of HEPA filters to remove smoke/chemical aerosols [29–31]. Our method of aerosolizing a live virus of similar size to SARS-CoV-2 has several advantages over smoke/chemical methods. First, our method detects and quantifies viable viruses still able to infect *E. coli*. Second, our method allows quantification of viruses settling on surfaces, infiltrating PPE, and most importantly depositing in a human upper airway.

We demonstrated 2 significant effects of HEPA filtration on virus-laden aerosol. First, relating to HCWs, we found that a HEPA filter enhances the PPE effectiveness so that a quantitatively fit-test_PASSED_ N95 mask provided almost complete protection against skin under the mask and nasal contamination from virus aerosol. The level of protection offered from nasal virus deposition is critical as it directly relates to possible ports of entry of SARS-CoV-2 including via the upper respiratory tract and the lungs. Although particle deposition in the respiratory tract is complex, an extensively verified model for regional aerosol deposition shows that for molecules in the range 0.2–10 µm, deposition in the anterior nose is greater than in the bronchioles when breathing through either the mouth or nose [32, 33]. As such, the finding in our study of almost complete protection from virus aerosol on nasal swab can be used to infer almost complete protection from lung deposition. Second, we found that HEPA filter deployment reduced virus counts on room settling plates compared to the no HEPA filter condition. However, there was still extensive environmental virus contamination with the HEPA filter deployed. This has clear implications for the deployment of such devices in hospital environments. Although the HEPA filter reduced aerosol load and therefore likely reduces risk, it does not negate environmental contamination in the same way as our previously published combined patient hood/HEPA filter “point of emission” strategy [20].

There are several limitations to this study. First, this was an experimental study using high quantities of a marker virus rather than a clinical observational trial. Therefore, we would strongly caution against making direct quantitative comparisons (based on absolute PFU values in each mask condition) against published minimum infective doses for respiratory viruses. This experimental design does not allow us to directly determine whether virus counts in HCWs’ nares may result in clinical infection. Similarly, the HCW remained seated in the clinical room for the entire exposure period. While this provided good experimental control of exposure, this behavior is not directly generalizable to clinical practice. Second, we aerosolized a higher viral load (10^8^/mL) compared to aerosols generated by patients infected with coronaviruses [34]. However, the choice of bacteriophage titer was determined based on detection sensitivity experiments (for skin swab and settle plates) that would allow us to assess relative reductions between mask variants. Importantly, the viral load used was similar to previous experiments by our group [20] and others [35] using bacteriophage methodologies. Importantly, the use of high viral load provides an appropriately strong safety test of these PPE strategies. Third, we observed significant variability in viruses quantified from swab samples. Our analyses demonstrate this variability is driven by between-day differences, likely resulting from differences in bacteriophage titer aerosolized on a given day. Our within-day data demonstrate consistent relative reductions in virus counts between the fitted N95 mask and the control (Figure 2*A* and 2*B*). This is important given that a high degree of variability in the viral load/exposure is an expected phenomenon in healthcare settings. Importantly, for our main finding of the effect of quantitative fit-test_PASSED_ N95 and HEPA filter, the results indicating almost universal zero detectable virus are reassuring in this regard. Fourth, our experiments are conducted in a sealed clinical room as opposed to a standard clinical room, which should have approximately 6–12 air exchanges per hour. Therefore, no mixing between clean/external air was occurring (eg, ingress under door gap or via ventilation openings). The lack of mixing biases toward a higher aerosol load in the experimental room and thus represents a worse-case scenario for room ventilation. This again provides reassurance about the high effectiveness of the combination of fit-test_PASSED_ N95, PPE, and HEPA filter. Finally, although particle analysis was not performed during conditions, indicative reference recordings in our experiments demonstrate a high particle load of size distribution in the range generated by humans [35], confirming the performance of the Pari-PEP nebulizer.

In conclusion, the emergence of more transmissible variants of SARS-CoV-2 have highlighted the gaps in protecting HCWs that were exposed in 2020–2021. Healthcare providers must deploy a simultaneous array of mitigation strategies to optimize HCW safety. N95 masks that pass a quantitative fit-test combined with a HEPA filter can offer protection against high virus aerosol loads, at close range, for prolonged periods of time.

## Supplementary Material

jiac195_Supplementary_Data

## Supplementary Data

Supplementary materials are available at *The Journal of Infectious Diseases* online (http://jid.oxfordjournals.org/). Supplementary materials consist of data provided by the author that are published to benefit the reader. The posted materials are not copyedited. The contents of all supplementary data are the sole responsibility of the authors. Questions or messages regarding errors should be addressed to the author.

## Notes

***Author contributions.*** Conceptualization and formal analysis: S. A. L., S. A. J. Funding acquisition: S. A. J., S. A. L., B. A. E., G. S. H., D. M. Investigation: S. A. L., D. S., J. J. B., S. D., D. M. K., S. A. J. Methodology: S. A. L., D. S., J. J. B., S. A. J. Project administration: S. A. J. Resources: J. J. B., B. A. E., S. A. J. Validation: S. A. L., D. S., J. J. B., S. D., D. M. K., S. A. J. Visualization: S. A. L. All authors performed writing and editing of the manuscript.

***Data sharing.*** Additional methodologic details, supplementary analyses, and links to the study data are located in the Supplementary Data.

***Financial support.*** This work was funded by an Epworth Hospital Capacity Building Research Grant (grant number EH2020-654).

***Potential conflicts of interest.*** All authors: No reported conflicts of interest.
